# Mitochondria of Porcine Oocytes Synthesize Melatonin, Which Improves Their In Vitro Maturation and Embryonic Development

**DOI:** 10.3390/antiox13070814

**Published:** 2024-07-07

**Authors:** Tianqi Zhu, Laiqing Yan, Shoulong Deng, Wenkui Ma, Fan Xia, Likai Wang, Xiao Ma, Guangdong Li, Zixia Shen, Yiwei Wang, Yao Fu, Pengyun Ji, Bingyuan Wang, Lu Zhang, Guoshi Liu

**Affiliations:** 1 State Key Laboratory of Animal Biotech Breeding, Key Laboratory of Animal Genetics, Breeding and Reproduction of the Ministry of Agriculture, College of Animal Science and Technology, China Agricultural University, Beijing 100193, China; 2 National Center of Technology Innovation for Animal Model, National Health Commission of China (NHC) Key Laboratory of Comparative Medicine, Institute of Laboratory Animal Sciences, Chinese Academy of Medical Sciences and Comparative Medicine Center, Peking Union Medical College, Beijing 100021, China

**Keywords:** porcine oocyte, mitochondria, melatonin, electron transport chain, in vitro maturation

## Abstract

The in vitro maturation efficiency of porcine oocytes is relatively low, and this limits the production of in vitro porcine embryos. Since melatonin is involved in mammalian reproductive physiology, in this study, we have explored whether endogenously produced melatonin can help in porcine oocyte in vitro maturation. We have found, for the first time in the literature, that mitochondria are the major sites for melatonin biosynthesis in porcine oocytes. This mitochondrially originated melatonin reduces ROS production and increases the activity of the mitochondrial respiratory electron transport chain, mitochondrial biogenesis, mitochondrial membrane potential, and ATP production. Therefore, melatonin improves the quality of oocytes and their in vitro maturation. In contrast, the reduced melatonin level caused by siRNA to knockdown *AANAT* (siAANAT) is associated with the abnormal distribution of mitochondria, decreasing the ATP level of porcine oocytes and inhibiting their in vitro maturation. These abnormalities can be rescued by melatonin supplementation. In addition, we found that siAANAT switches the mitochondrial oxidative phosphorylation to glycolysis, a Warburg effect. This metabolic alteration can also be corrected by melatonin supplementation. All these activities of melatonin appear to be mediated by its membrane receptors since the non-selective melatonin receptor antagonist Luzindole can blunt the effects of melatonin. Taken together, the mitochondria of porcine oocytes can synthesize melatonin and improve the quality of oocyte maturation. These results provide an insight from a novel aspect to study oocyte maturation under in vitro conditions.

## 1. Introduction

Melatonin, also known as N-acetyl-5-methoxy tryptamine, is widely present in almost all organisms. In mammals, all tissues and organs, especially the pineal gland, convert tryptophan to 5-hydroxy tryptophan by hydroxylase; this intermediate is then metabolized into 5-hydroxytryptamine (5HT) by decarboxylase. This 5-HT is then catalyzed by arylamine N-acetyltransferase (SNAT/AANAT) into N-acetylserotonin. Finally, N-acetylserotonin is converted to melatonin by hydroxyindole-O-methyltransferase (HIOMT/ASMT). The melatonin is synthesized by the pineal gland and directly released into the cerebrospinal fluid and blood [1,2,3], while the extra pineal synthesized melatonin is primarily intended for local utilization (autocrine and paracrine effects) [4]. The majority of melatonin is produced in the mitochondria [5] and it plays an important role in reducing oxidative stress. Our group is the first to report that the mitochondria of mouse oocytes synthesize melatonin [6].

Mitochondria are one of the most important organelles of oocytes. They are the powerhouses of cells and also control intracellular calcium homeostasis [7,8]. In addition, mitochondria play a central role in other functions, including the regulation of cell death and signaling pathways, iron metabolism, and the biosynthesis of certain organic compounds [9,10,11]. During folliculogenesis, the follicle undergoes substantial growth, expanding approximately 500-fold. This also makes oocytes within the follicle undergo major structural and biochemical transitions, including two meiotic divisions. To cope with such an energy-consuming process, the quantity and quality, as well as the distribution pattern, of mitochondria in the oocytes are also required to change [12].

The maturation of oocytes requires large amounts of ATP for their continued transcription and translation; therefore, this process needs sufficient numbers of functional mitochondria for ATP production. The fact is that the immature oocytes have limited mitochondrial activity and they depend upon the surrounding cumulus and granulosa cells to provide additional energy to support their maturation [13]. During the process of ovulation, the oocytes lose their connection to the cumulus cells, and this forces them to activate their own mitochondria. This is the reason why the matured oocytes have accumulated a sufficient number of mitochondria to generate ATP. Therefore, the ATP levels are elevated during polar body expulsion. Higher ATP levels are associated with higher fertilization rates in matured oocytes [14], while the lower ATP content in oocytes is associated with poor oocyte quality and lower levels of fertilization [15,16].

As mentioned above, melatonin is synthesized in mitochondria. Thus, melatonin is considered to have a major impact on mitochondrial functions, including increasing the efficiency of the electron transport chain [17], ATP production [18], and reducing oxidative damage to the mitochondria [19,20,21]. The oxidative damage caused by excess reactive oxygen species (ROS) impairs cellular function, leading to enzyme inactivation, lipid peroxidation, ATP depletion, and mitochondrial disturbance. It has been found that high levels of ROS and low antioxidant activity in the follicular fluid result in poor pregnancy outcomes after IVF (in vitro fertilization). Increased ROS levels during in vitro oocyte maturation are associated with chromosomal errors and the low developmental potential of oocytes [22,23]. For example, the increased ROS levels in cultured mouse oocytes alter the chromosomal arrangement of microtubules and spindles and inhibit their maturation [5]. Melatonin, as a potent antioxidant, can directly scavenge toxic oxygen derivatives [24,25] and stimulate the activities of antioxidant enzymes [26], including glutathione peroxidase (GSH-Px) and superoxide dismutase (SOD) [27]. However, whether porcine oocytes can synthesize melatonin and, if so, the effects of this endogenously generated melatonin on oocyte maturation are still unclear.

In this study, we aimed to explore the subcellular localization of melatonin synthesis in porcine oocytes and the role of locally synthesized melatonin in oocyte maturation. To achieve this purpose, interfering RNA has been used to knock down the expression of melatonin. The synthetic gene of *AANAT* was also used to explore further whether melatonin is involved in the maturation of porcine oocytes under in vitro conditions.

## 2. Materials and Methods

### 2.1. Ethics Statement

All animal studies followed the guidelines of the Animal Care and Use Committee of China Agricultural University and were approved by the Ethics Committee of the Agriculture University of China (permission number: AW01602202-1-6).

### 2.2. Chemicals

All chemicals used in this study were purchased from the Sigma-Aldrich Chemical Company (St. Louis, MO, USA), unless otherwise indicated.

### 2.3. The Procedure of In Vitro Porcine Oocyte Maturation

The ovaries of sows (donated by a local slaughterhouse, Beijing Food Company, Beijing, China) were collected and packed in thermostable containers (37 °C) with sterilized saline, penicillin, and streptomycin, then the samples were transported to the laboratory within 2 h and, finally, washed with 37 °C sterilized physiological saline. Thereafter, follicular fluid was extracted from the follicles (3–6 mm in diameter) with a syringe fitted with a 20 G needle. The cumulus-oocyte complex (COCS) was rinsed twice in HEPES-buffered lactate (TL-HEPES) medium and 3 times in hormone-free maturation medium. The COCs were then transferred into the maturation medium (50 oocytes per 0.5 mL of medium), which consisted of TCM-199 with 0.57 mM cysteine, 3.05 mM D-glucose, 0.91 mM sodium pyruvate, 10 ng/mL epidermal growth factor (EGF), 0.5 IU/mL luteinizing hormone (human origin, LH, sigma-L6420), 0.5 IU/mL follicle stimulating hormone (human origin, FSH, sigma-F4021), 0.1% polyvinyl alcohol (PVA), 75 mg/mL penicillin, 50 mg/mL streptomycin, 20 ng/mL LIF, 20 ng/mL IGF1, and 40 ng/mL FGF2 incubated at 38.5 °C, under 5% CO_2_ and in 100% humidity for 42–44 h for maturation. The maturated COCs were transferred to a culture medium containing 1 mg/mL hyaluronidase in TL-HEPES, then the cumulus cells were removed by vertexing and washed with TL-HEPES. These denuded oocytes were then used in the subsequent experiments [28].

### 2.4. Parthenogenetic Activation of Oocytes

The denuded porcine oocytes were activated in the activation medium (0.3 m mannitol, 0.05 mm CaCl_2_, 0.1 mm MgCl_2_, and 0.1% bovine serum albumin (BSA)) by an electrical pulse of DC 130 V/mm for 80 µs, using a BTX Electro-Cell Manipulator 2001 (BTX, Inc., San Diego, CA, USA). The activated oocytes were then rinsed in porcine zygote medium-3 (PZM-3) and cultured in a medium containing 5 µg/mL of cytochalasin B at 38.5 °C and 5% CO_2_ in air with 100% humidity for 5–6 h. The experiment was divided into 3 groups: the Control, 5-HT, and 5-HT + Lu groups.

### 2.5. In Vitro Culture (IVC) of Embryos

The parthenogenetically activated oocytes (approximately 20–30 oocytes per group) were placed in 100 µL droplets of PZM-3, supplemented with 0.6 mg/mL of BSA, and were incubated at 39 °C, 5% CO_2_, and 5% O_2_. The cleavage rate and blastocyst rate were observed and recorded after 48 and 168 h of IVC, respectively.

### 2.6. Calculation of Cumulus Cell Expansion and Polar Body Extrusion Rates in Porcine Oocyte Maturation

The expanded oocytes of matured COCs after 44 h of incubation were counted under the microscope. The expanded oocytes served as the numerator to divide the total number of oocytes in each well and to calculate the expansion rate of cumulus cells. Then, the cumulus cells were removed by the use of 0.3 mg/mL of hyaluronidase. The oocytes with discharged polar bodies were selected under microscopy with a 20-times eyepiece. The polar body discharge rate was calculated against the total number of oocytes in each well.

### 2.7. Cortical Granule Migration Assay

The zona pellucida of MI-stage oocytes was removed with 0.1% pronase; the oocytes were washed 3 times with PBS and incubated in a CO_2_ incubator to restore them to a normal shape. Then, they were fixed with 4% formaldehyde at room temperature for 30 min and the oocytes were washed 3 times with blocking solution for 5 min each time; the blocking solution was PBS + 3 mg/mL BSA + 7.5 mg/mL glycine. The blocked oocytes were infiltrated with 0.5% Triton-X100-PBS-0.1% PVA for 30 min, then incubated at room temperature for an additional 30 min in dark conditions and stained with a staining solution of 100 μg/mL PBS-FITC-PNA (SigmaL-7381). The samples were washed with PBS 3 times, placed on a glass slide, and covered with a paraffin-coated cover glass. The distribution of oocyte cortical granules was observed under a laser confocal microscope.

### 2.8. Mitochondrial Levels and Their Distribution in Porcine Oocytes

The oocytes were vortexed to remove the zona pellucida to obtain naked oocytes. The mitochondria were labeled with MitoTracker Red CMXRos for 30 min (PBS washing solution + 500 nmol/L MitoTracker Red CMXRos), mounted onto slides, and analyzed under a fluorescence microscope.

### 2.9. Subcellular Localization of AANAT, Detected by Immunoelectron Microscopy

Approximately 1000 oocytes were collected for fixation with paraformaldehyde. The fixed samples were washed with PBS to remove the glutaraldehyde residuals and dehydrated through a 30, 50, 75, 85, 95, and 100% alcohol gradient, respectively, in sequence and with xylene in the final stage. The samples were then soaked in epoxy resin, fixed, and embedded into blocks by temperature gradient treatment in an oven. The block was then trimmed with a razor blade and any obvious follicle structure on the surface of the ovary was removed. The trimmed samples were sliced with an automatic microtome in sequence. The slice thickness was 100 nm. The slices containing the oocyte structure were selected and fixed on the copper grid. The AANAT antibody was diluted to 1:100 and made into 30 μL small droplets, then the sample was submerged into the droplets, pre-incubated at room temperature for 1 h, and then incubated at 4 °C overnight. Thereafter, the samples were fully washed to remove the primary antibody and then incubated with 30 μL of gold-labeled secondary antibody (diluted 1:2000) at room temperature for 2 h. After washing away the secondary antibody, the samples were incubated in uranyl acetate for 15 min. The samples were analyzed under an electron microscope and then photographed.

### 2.10. Lipid Droplet Staining of Porcine Oocytes

After 44 h of maturation, the COCs were harvested from the IVM medium, then the cumulus cells around the oocytes were removed and stained with 20 μg/mL BODIPY 493/503 (Thermo, Waltham, MA, USA, D3922). The stained MII oocytes were then placed in a glass petri dish and observed under a confocal microscope with image-taking (Nikon A1HD25, Tokyo, Japan). The excitation wavelength was 405 nm for LipiBlue and 488 nm for BODIPY 493/503. An NIS (Nikon) was used to take pictures and to calculate the fluorescence intensity of the lipid droplets.

### 2.11. Mitochondrial Membrane Potential Analysis with JC-10 Staining

JC-10 is a fluorescent probe used for detecting mitochondrial membrane potential, ΔΨm. When the mitochondrial membrane potential is high, JC-10 gathers in the mitochondrial matrix to form a polymer with red fluorescence; when the mitochondrial membrane potential is low, JC-10 is a monomer with green fluorescence. The oocytes were incubated with the diluted (200XJC-10 to 1X) JC10 solution at 37 °C for 20 min, then washed with JC-10 staining buffer 3 times, after which the samples were placed in the covered slides and observed under a laser confocal microscope. Changes in mitochondrial membrane potential are detected by fluorescent color shifts. The relative ratio of red–green fluorescence is commonly used to measure the ratio of mitochondrial depolarization.

### 2.12. Procedure of Immunofluorescence Staining

The oocytes were fixed in 4% paraformaldehyde (PFA) at room temperature for 45 min and washed with PBS-0.1%PVA 3 times, for 10 min each time. Hole punching in the cell membrane was achieved with 0.5%Triton-X 100-PBS-0.1% PVA incubation at room temperature for 1 h. First, the samples were blocked in 3% BSA-0.1%Triton-X 100-PBS-0.1% PVA solution for 1 h at room temperature; then, the MII oocytes were incubated with AANAT antibody (1:100) and ASMT (1:100) antibody (diluted in sealing solution) at 4 °C for 12 h, and washed with PBS-0.1% PVA for 3 times, for 10 min each time. Then, they were incubated with the secondary antibody (1:200) at room temperature in the dark for 1 h, washed with DPBS-0.1%PVA 3 times, for 20 min each time; Hoechst33342 was used to stain the nuclei. The samples were mounted as slices, observed under a laser confocal microscope, and photographs were taken.

### 2.13. Melatonin Assay with High-Performance Liquid Chromatography (HPLC)-Tandem Mass Spectrometry

First, 200 μL of mitochondrial culture solution was mixed with 800 μL methanol and centrifuged at 12,000 r/min, at 4 °C for 20 min. The samples were filtered with a 2-um filter. The sample was injected into a HPLC-tandem mass spectrometry system. For the HPLC detecting system, the mobile phase was formulated with solutions A (0.1% formic acid solution) and B (methanol). A gradient elution procedure was carried out in the order of 10% B-phase elution for 0–1 min, 60% B-phase for 2–3.5 min, and 10% B-phase for 3.5–5 min. The flow rate of the mobile phase was 0.4 mL/min. A temperature of 40 °C was chosen for the column. The injected sample volume was 2 μL. The MS/MS system comprised a triple quadrupole mass spectrometer with electrospray ionization (ESI). The positive mode acquisition is used to collect MS/MS data. Multiple reaction monitoring (MRM) was used to identify MT and MT-d4. The gas temperature and flow rate were maintained at 350 °C and 6 L per minute, respectively. The nebulizer pressure was 50 psi, and the capillary voltage setting was 3500 V. The sheath gas heater reached 300 °C, with the flow of sheath gas at 10 mL/min. MT’s production ions (*m*/*z*) were 174.2 and 159.1, while its precursor ion (*m*/*z*) was 233.1. The collision energy at 10 V was 233.1 > 174.1. The collision energy at 25 V was 233.1 > 159.1. MT-d4’s precursor ion (*m*/*z*) was 237.1, while its production ions (*m*/*z*) were 163.1 and 178.2. Where 237.1 > 178.2 and 237.1 > 163.1, the collision energy was 25 V. Every fragmentor above this was 75 V.

### 2.14. Assay of ATP

The zona pellucida of the oocytes were removed with pronase and the cleaned oocytes were washed 3 times with TL solution; 12 oocytes were transferred into 50 μL lysate solution, vortexed until they were fully lysed, and the samples were kept at 4 °C or on the top of the ice for a short period. A 96-well light-proof enzyme labeling plate was used for the study. First, 50 μL of ATP detection solution was added to the standard and sample wells of this plate, respectively, and left at room temperature for 5 min to consume the background ATP; then, the lysed samples were added to the preprepared wells of the plate and mixed well. An Infinite F200 microplate reader was used to detect the ATP content. The ATP content of each sample well was calculated based on the ATP standard curve, and the value was divided by the number of oocytes to obtain the ATP content of each oocyte.

### 2.15. RNA Interference Assay

The porcine *AANAT* gene was used as a template to design interfering RNA by the Suzhou Gemma Gene Co., Ltd., Suzhou, China, and the designed sequence was compared with BLAST to exclude homology with other genes. The final designed porcine siRNA sequence is as follows:

siAANAT: sense(5′-3′): GGGACUGAAAUAAAGAGAUTT;

antisense(5′-3′): AUCUCUUUAUUUCAGUCCCTT.

The cumulus oocytes were removed from the cumulus granulosa cells, and 10 plsiRNA (20 μM) was injected into the cytoplasm of each oocyte using a micromanipulator [29]. The experiment was divided into 3 groups: siNC, siAANAT, and siAANAT + MT groups, respectively.

### 2.16. Real-Time Fluorescent Quantitative PCR

The oocytes extracted from COCs were washed 3 times with PBS and stored at 80 °C until RNA extraction. Total RNA was extracted using TRIzol (Invitrogen Inc., Carlsbad, CA, USA), quantified by measuring the absorbance at 260 nm, and stored at −80 °C until it was assayed. The mRNA levels of the relevant genes were assessed in LightCycler (Roche Applied Science, Mannheim, Germany) by quantitative RT-PCR using the OneStep SYBR PrimeScript RT-PCR kit (Takara Bio. Inc., Tokyo, Japan). After melting curve analysis, the accumulated level of fluorescence was analyzed by the second derivative method, then the expression level of the target gene in each sample was normalized to that of β-actin. The primer pairs for mRNAs are shown in Table 1.

### 2.17. Statistical Analysis

Unless otherwise specified, the data are expressed as mean ± SME. An analysis of variance (ANOVA) was used to analyze the normality among the groups, followed by Dunnett’s test. All tests were performed by SPSS26.0 statistical software. *p* < 0.05 denoted a significant difference.

## 3. Results

### 3.1. The Capacity of Mitochondria in Porcine Oocytes on Melatonin Biosynthesis during Their In Vitro Maturation

**Figure 1:**
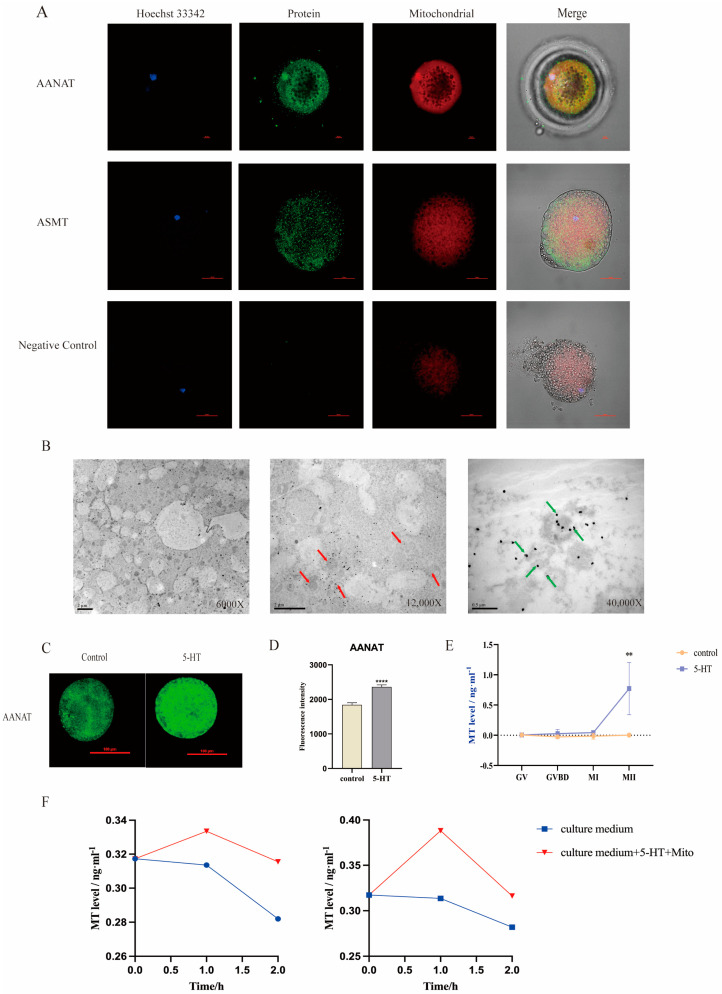
Melatonin synthesis in mitochondria during the process of the in vitro maturation of porcine oocytes. (**A**) Immunofluorescent staining of AANAT and ASMT in porcine oocytes. Scale bar: 50 µm. (**B**) AANAT subcellular distribution in porcine oocytes; red arrows point to mitochondria and green arrows point to AANAT synthetase. (**C**) Immunofluorescent staining of AANAT in porcine oocytes with 5HT treatment; scale bar: 100 µm. (**D**) Statistical analysis of AANAT level of oocytes (n = 25). (**E**) Melatonin levels in the culture medium of oocytes at each mature stage; n = 8. (**F**) Melatonin levels in the mitochondrial culture medium of granulosa cells (**left**) and oocytes (**right**); n = 3. ** *p* < 0.01, **** *p* < 0.0001.

By the use of immunofluorescence staining and confocal microscopy, both the synthetic melatonin enzymes AANAT and ASMT were found to be expressed in oocytes and colocalized with mitochondria (Figure 1A). The immunoelectron microscopy results confirmed that a major portion of AANAT was distributed in mitochondria but some was also present in the cytoplasm (Figure 1B). In order to explore whether melatonin is synthesized during the process of maturation of porcine oocytes, 5HT, a precursor of melatonin, was added to the maturation medium. Then, the medium was collected for melatonin assay by ultra-high performance liquid chromatography–tandem mass spectrometry (UPLC-MS/MS) The results showed that during the process of maturation, the level of melatonin gradually increased and was then sharply elevated from the MI to MII stages, compared to the control group (*p* < 0.01) (Figure 1E). Accordingly, after adding 5-HT, the protein level of AANAT was also significantly increased compared to the control group (*p* < 0.01) (Figure 1C,D). To further explore the subcellular sites of melatonin synthesis, the mitochondria of oocytes and cumulus cells were extracted and incubated with 5-HT. The culture fluid was collected at 0, 1, and 2 h, respectively, for melatonin detection. The results showed that melatonin was detected in the mitochondrial culture medium. The melatonin production that was extracted from the mitochondria of oocytes supplemented with 5-HT significantly increased compared to the controls after 1 h of incubation (Figure 1F). These results indicate that the mitochondria of porcine oocytes synthesize melatonin during in vitro maturation.

### 3.2. 5-HT Supplementation Improves the Quality of Porcine Oocytes

**Figure 2:**
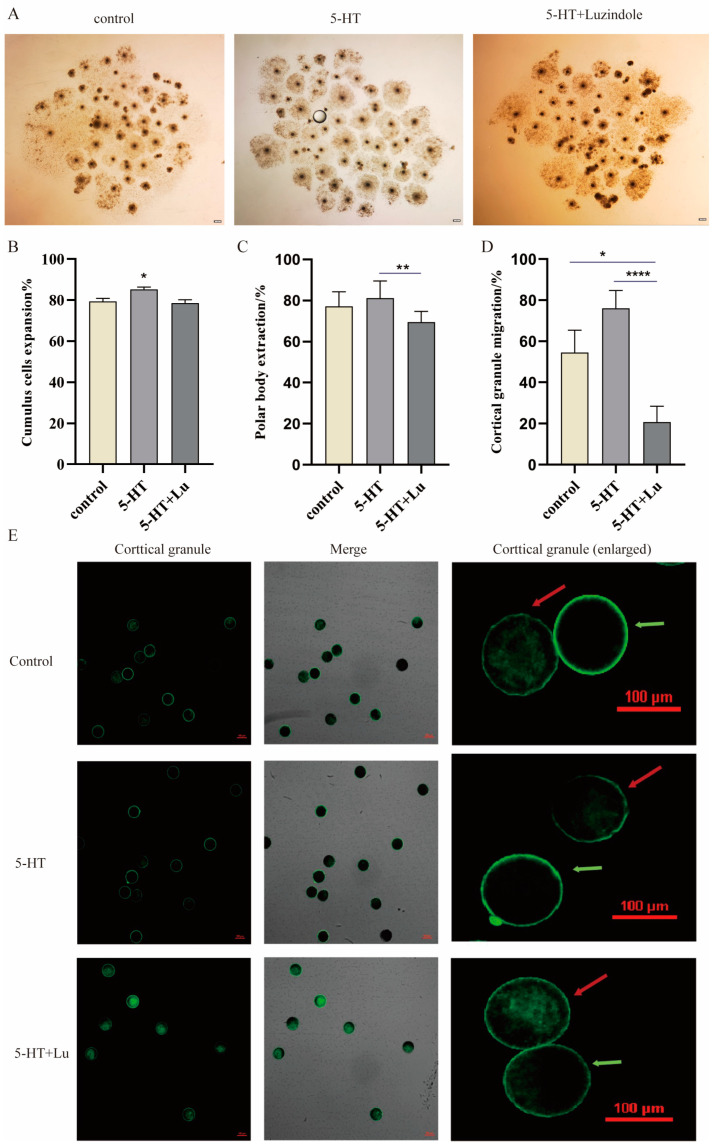
The effects of 5-HT and Luzindole on the maturation of porcine oocytes. (**A**) Cumulus cell expansion of oocytes after 44 h of maturation, scale: 200 μm. (**B**) The statistical analyses of cumulus cell expansion; Control (n = 789), 5-HT (n = 741), and 5-HT + Lu (n = 686). (**C**) The statistical analyses of polar body expulsion; Control (n = 570), 5-HT (n = 596), and 5-HT + Lu (n = 391). (**D**) The statistical analyses of cortical granule migration; Control (n = 22), 5-HT (n = 25), and 5-HT + Lu (n = 29). (**E**) Fluorescence staining of cortical granules; green arrows indicate normal migration and red arrows indicate abnormal migration. Scale bar: 100 μm. * *p* < 0.05, ** *p* < 0.01, and **** *p* < 0.0001.

Since 5-HT could increase melatonin production in oocytes, 5-HT was added to the COCS maturation medium. The results showed that 5-HT significantly increased the expansion rate of porcine cumulus cells (*p* < 0.05) (Figure 2A,B) and the normal migration of cortical granules (*p* < 0.05) (Figure 2D,E), compared to the control group. While the addition of Luzindole, a melatonin receptor inhibitor, significantly reduced the normal mobility of cortical granules (*p* < 0.001) and the cumulus expansion rate (*p* < 0.05) (Figure 2B), compared to the control group, Luzindole also reduced the polar body excretion rate, but this decrease was not significantly different compared to the control group (*p* > 0.05) (Figure 2C). All the results indicated the promotive effects of melatonin on both the nuclear and cytoplasmic maturation of porcine oocytes, and these activities might be partially mediated by melatonin receptors.

### 3.3. Effects of 5-HT Supplementation on the Distribution and Function of Mitochondria in Oocytes

**Figure 3:**
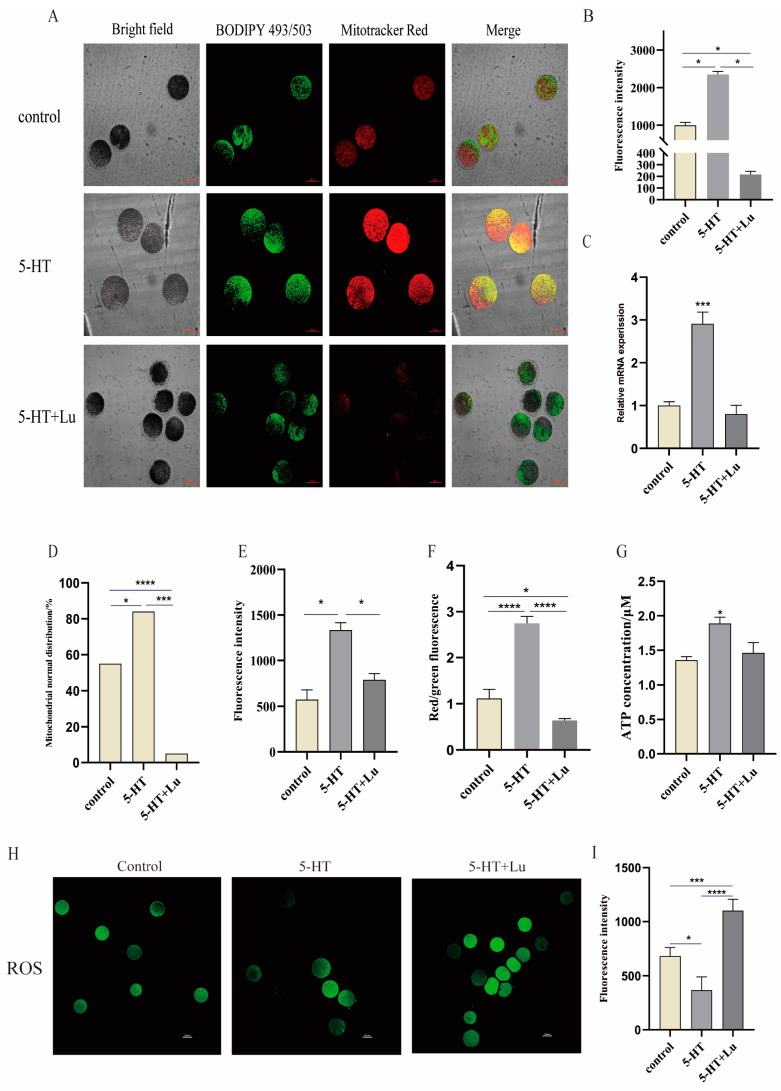
Effects of 5HT on mitochondrial function in porcine oocytes. (**A**) Lipid droplet and mitochondrial staining images of oocytes. (**B**) Statistical analysis of Mitotracker Red fluorescence staining intensity; Control (n = 20), 5-HT (n = 18), and 5-HT + Lu (n = 18). (**C**) Relative SIRT1 mRNA level, n = 4. (**D**) Mitochondrial distribution; Control (n = 20), 5-HT (n = 19), and 5-HT + Lu (n = 17). (**E**) BODIPY493/503 fluorescence staining intensity; Control (n = 19), 5-HT (n = 18), and 5-HT + Lu (n = 19). Scale bar: 100 μm. (**F**) Mitochondrial membrane potential. (**G**) ATP level of porcine oocytes, n = 36. (**H**) ROS staining images of porcine oocytes. (**I**) ROS levels in oocytes; Control (n = 22), 5-HT (n = 31), and 5-HT + Lu (n = 34). Scale bar: 100 μm. * *p* < 0.05, *** *p* < 0.001, and **** *p* < 0.0001.

To explore the effect of endogenously produced melatonin on mitochondrial properties in oocytes, 5-HT was supplied to the cell culture medium to improve the melatonin production of oocytes. The result showed that this treatment significantly increased the mitochondrial density (*p* < 0.05) (Figure 3A,B) and the normal distribution of oocytes compared to the control group (*p* < 0.05). Accordingly, the mitochondrial biogenesis-related gene SIRT1 was significantly upregulated compared to the controls (Figure 3C). Again, the melatonin receptor inhibitor, Luzindole, significantly blunted all these beneficial alterations of mitochondria (*p* < 0.05) (Figure 3B,D), indicating that the beneficial effects of melatonin are mediated by its receptor. It is worth noting that the 5-HT supplementation also increased the number of lipid droplets in porcine oocytes compared to the control (*p* < 0.05), while Luzindole reduced this increase (*p* < 0.05) (Figure 3A,E).

**Figure A1:**
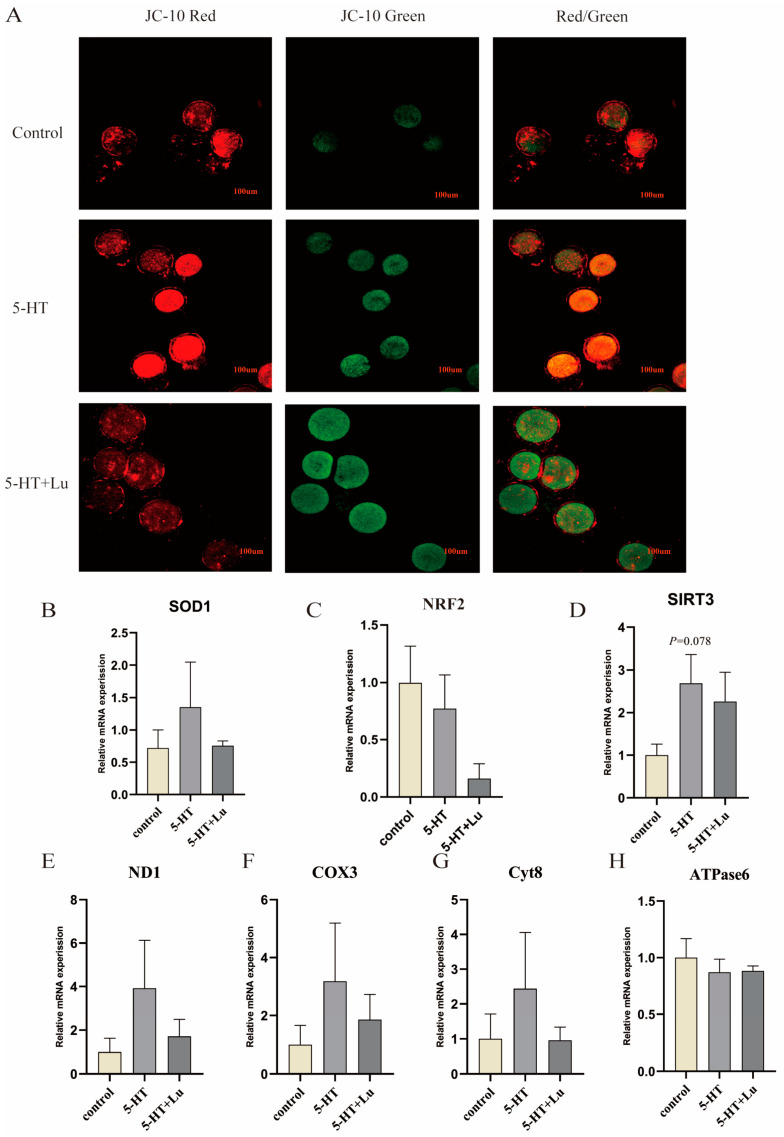
Effects of 5-HT on mitochondrial membrane potential of porcine oocytes. (**A**) JC-10 staining images of pig oocyte mitochondria. (**B**) Relative SOD1 mRNA expression level; n = 4. (**C**) Relative NRF2 mRNA expression level; n = 4. (**D**) Relative SIRT3 mRNA expression level; n = 4. (**E**–**H**) The mRNA levels of mitochondrial complex I (ND1), complex III (COX3), complex IV (CytB), and complex V (ATPase6); n = 4.

To explore whether endogenously generated MT promotes the energy metabolism of oocytes, the oocytes were stained with JC-10 fluorescence and the effect was analyzed with a confocal microscope (Figure A1A). The results showed that 5-HT supplementation significantly increased the mitochondrial membrane potential (*p* < 0.0001), as indicated by increased JC-10 fluorescence intensity, and also the ATP content of oocytes compared to the control (*p* < 0.05). Luzindole significantly blunted the mitochondrial membrane potential (*p* < 0.0001) and the ATP content (*p* < 0.05) was increased by 5-HT (Figure 3G–F). At the molecular level, 5-HT treatment increased the activities of mitochondrial complex Ⅰ (ND1), complex Ⅲ (COX3), and complex Ⅳ (CytB), but the differences did not reach statistical significance, while complex Ⅴ (ATPase6) showed little change compared to the control (Figure A1E–H). The 5-HT treatment upregulated the gene expressions of SIRT3 and SOD1 (Figure A1B–D) and significantly decreased the ROS level in oocytes (*p* < 0.05), but this decrease was blunted by Luzindole (*p* < 0.001), which hindered the antioxidant effect of endogenous melatonin (*p* < 0.0001) (Figure 3H,I).

### 3.4. Effects of siAANAT on the Quality and Maturation of Porcine Oocytes

**Figure 4:**
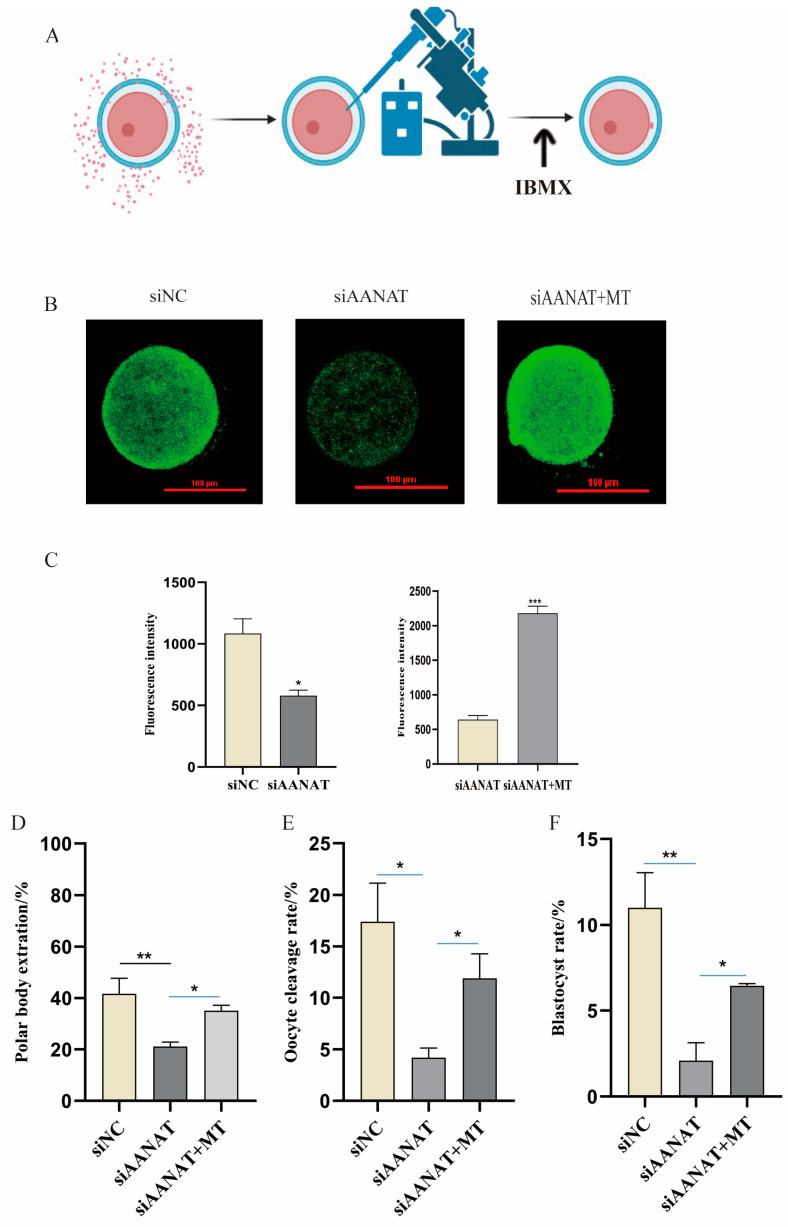
Effects of siAANAT on the maturation quality of porcine oocytes. (**A**) siRNA flowchart. (**B**) Immunofluorescence staining of AANAT protein in pig oocytes. Scale bar: 100 μm. (**C**) Statistical analysis of the fluorescence intensity of the AANAT protein. siNC (n = 39), siAANAT (n = 23), and siAANAT + MT (n = 19). (**D**) Polar body extraction rate. siNC (n = 355), siAANA T (n = 473), and siAANAT + MT (n = 254). (**E**) Cleavage rate. siNC (n = 125), siAANAT (n = 94), and siAANAT + MT (n = 93) groups. (**F**) Blastocyst rate. siNC (n = 125), siAANAT (n = 94), and siAANAT + MT (n = 93) groups. * *p* < 0.05, ** *p* < 0.01, *** *p* < 0.001.

siAANAT was designed to suppress the melatonin production in oocytes. siAANAT was microinjected into denuded oocytes in the GV stage and cultured in vitro for 44 h, then the MⅡ-stage oocytes were selected for immunofluorescence staining. The results showed that the siAANAT oocytes had significantly lower AANAT protein expression than that in siNC oocytes (*p* < 0.01), indicating that siRNA successfully knocked down the expression of AANAT in the oocytes. Then, the polar body excretion rate of the oocytes was counted. The results showed that the polar body excretion rate of oocytes in the siAANAT group was significantly lower than that in the control group (*p* < 0.01). Melatonin supplementation significantly increased the suppressed polar body excretion rate caused by siAANAT (*p* < 0.05) (Figure 4D). After the parthenogenetic activation of oocytes, it was found that the cleavage rate and blastocyst rate of the siAANAT group were significantly lower than those of the siNC group, while melatonin supplementation could improve the quality of porcine oocyte maturation and embryo development potential (*p* < 0.05) (Figure 4E,F).

### 3.5. Effects of siAANAT on Mitochondrial Distribution and ATP Production in Porcine Oocytes

**Figure 5:**
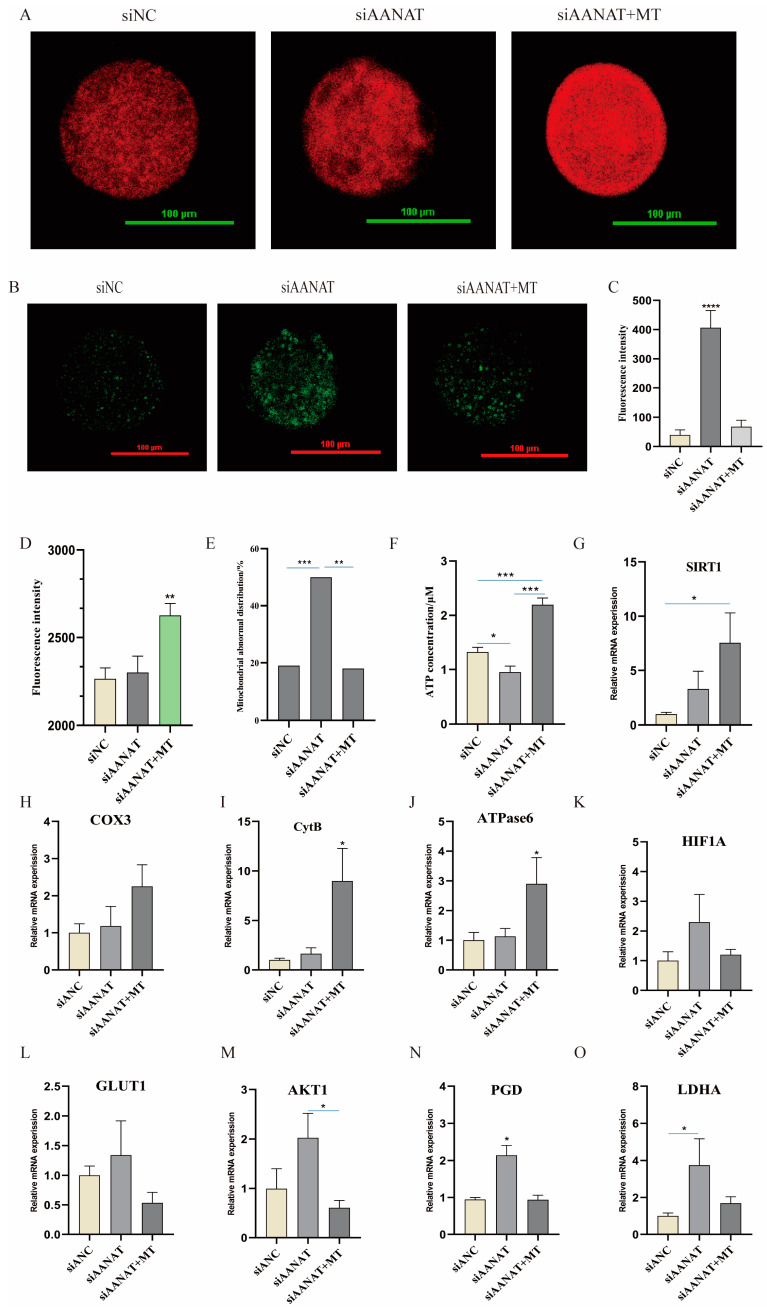
Effects of siAANAT on the metabolic patterns in porcine oocytes. (**A**) Fluorescence staining images of mitochondria in porcine oocytes. Scale: 100 μm. (**B**) ROS fluorescent staining in oocytes. (**C**) Statistic analysis of ROS fluorescence staining intensity in oocytes. SiNC (n = 6), siAANAT (n = 5), and siAANAT + MT (n = 8). (**D**) Fluorescence intensity of mitochondrial fluorescent staining. SiNC (n = 32), siAANAT (n = 17), and siAANAT + MT (n = 19). (**E**) Abnormal distribution ratio of mitochondria. siNC (n = 71), siAANAT (n = 34), and siAANAT + MT (n = 22) group. (**F**) ATP content. siNC (n = 48), siAANAT (n = 48), and siAANAT + MT (n = 48) group. (**G**) Relative SIRT1 mRNA expression level; n = 3. (**H**) Relative COX3 mRNA expression level; n = 3. (**I**) Relative CytB mRNA expression level; n = 3. (**J**) Relative ATPase6 mRNA expression level; n = 3. (**K**) Relative HIF1A mRNA expression level; n = 3. (**L**) Relative GLUT1 mRNA expression level; n = 3. (**M**) Relative AKT1 mRNA expression level; n = 3. (**N**) Relative PGD mRNA expression level; n = 3. (**O**) Relative LDHA mRNA expression level; n = 3. * *p* < 0.05, ** *p* < 0.01, *** *p* < 0.001 and **** *p* < 0.0001.

**Figure A2:**
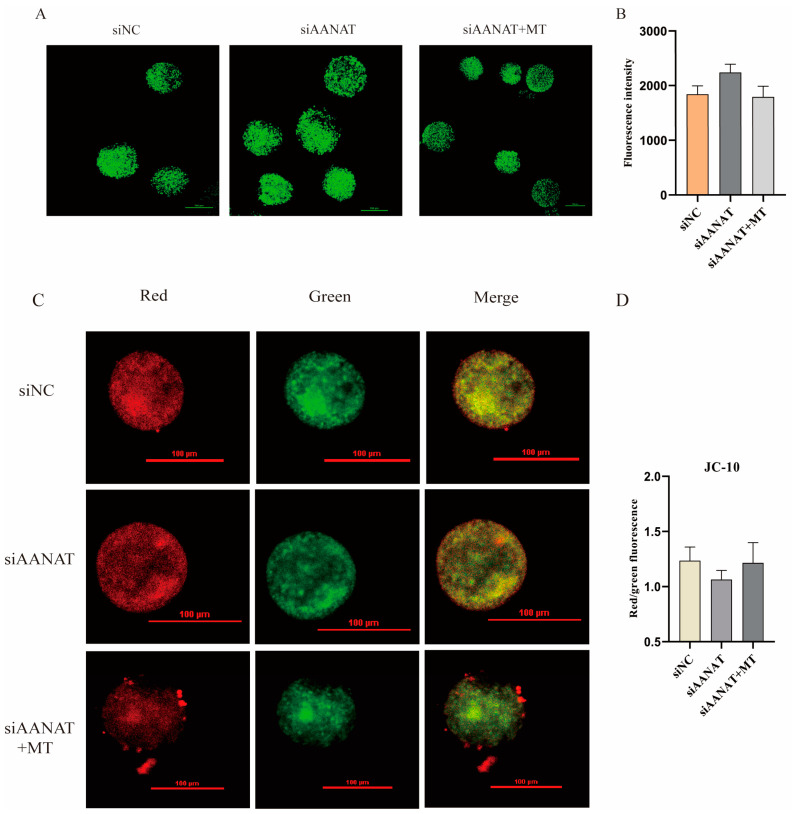
Effects of siAANAT on oocyte lipid and mitochondrial membrane potential levels after knockdown. (**A**) Fluorescent staining images of lipid droplets in porcine oocytes; scale: 100 μm. (**B**) Fluorescence intensity of siNC (n = 14), siAANAT (n = 6), and siAANAT + MT (n = 6) groups. (**C**) oocyte mitochondrial JC-10 staining; scale: 100 μm. (**D**) JC-10 fluorescence intensity of oocyte mitochondria in siNC (n = 4), siAANAT (n = 4), and siAANAT + MT (n = 3) groups.

Since the lipid is an important substrate of mitochondrial metabolism, the lipid levels in oocytes were measured. The results showed that the number of lipid droplets in siAANAT oocytes showed no significant difference compared to other groups (Figure A2A,B). The number of mitochondria in the siAANAT oocytes showed no significant difference with the siNC group but had a higher abnormal distribution rate than that in the siNC oocytes (*p* < 0.001) (Figure 5E). Interestingly, melatonin treatment rescued the mitochondrial abnormal distribution caused by siAANAT (*p* < 0.01), and also significantly increased the number of mitochondria (*p* < 0.01) (Figure 5A,D). In addition, the expression of SIRT1 in the melatonin treatment group was significantly upregulated compared to other groups (*p* < 0.05) (Figure 5G). At the same time, the ROS level in the siAANAT group was significantly increased (*p* < 0.0001), and was also reduced by melatonin supplementation (*p* < 0.0001) (Figure 5B,C). The mitochondrial membrane potential in the siAANAT oocytes was lower than that in the control group and in the melatonin-treated group, but the difference was not significant (*p* > 0.05) (Figure A2C,D). However, the ATP level in the siAANAT oocytes was significantly lower than that in the control and melatonin-treated oocytes (*p* < 0.05) (Figure 5F).

### 3.6. Effects of siAANAT on the Metabolic Pattern of Porcine Oocytes

In this study, we also found that melatonin slightly increased the expression of mitochondrial complex III (COX3) but significantly upregulated the expression of complex IV (CytB) and complex V in siAANAT oocytes compared to the other groups (*p* < 0.05) (Figure 5H–J). The results led us to wonder whether siAANAT makes oocytes mainly to produce energy through glycolysis. To further understand the effects of siAANAT on the metabolic pattern of porcine oocytes, key gene expression in the glycolytic pathway was measured. The results showed that the expressions of HIF1A and GLUT1 in siAANAT oocytes were slightly upregulated (Figure 5K,L); however, at the same time, the expressions of phospho-6-gluconate dehydrogenase (PGD) and lactate dehydrogenase (LDHA) were significantly upregulated compared to the control oocytes (*p* < 0.05) (Figure 5N,O). Melatonin supplementation significantly downregulated the expression levels of PGD (*p* < 0.05) and serine-threonine protein kinase 1 (AKT1) (*p* < 0.05) (Figure 5M,N).

## 4. Discussion

The maturation of mammalian oocytes requires substantial energy, and studies have shown that porcine oocytes consume glucose to support their final maturation [30]. Recently, evidence has emerged that lipids are a key nutrient and are even a major energy source for porcine oocytes [31]. Numerous studies have shown that in early embryo development, mitochondria are responsible for providing sufficient ATP for most cellular processes through oxidative phosphorylation. The number of mitochondria increases substantially during embryonic development to provide the energy required for blastocyst formation; therefore, mitochondrial dysfunction leads to developmental arrest in early embryos [32].

Evidence has shown that mitochondrial functions can be influenced by several factors, and melatonin is one of them. Melatonin can regulate mitochondrial functions by scavenging free radicals, activating uncoupling proteins, maintaining optimal mitochondrial membrane potential, and promoting mitochondrial biogenesis [25,33,34,35,36,37]. The activities of melatonin on the mitochondria may relate to its effect in promoting oocyte maturation, fertilization, and early embryonic development in mammals [38]. AANAT is the rate-limiting enzyme for melatonin synthesis [39]. Our previous study found that melatonin was synthesized in the mitochondria of mouse oocytes during its maturation [6]. In the current study, we confirmed that AANAT was co-localized with mitochondria in porcine oocytes, and 5-HT supplementation during the in vitro porcine oocyte maturation process significantly increased the melatonin production compared to the control group (*p* < 0.01). In addition, both the in vitro-cultured mitochondria isolated from cumulus cells and oocytes can release melatonin to the culture medium; with 5-HT supplementation, the mitochondria produced significantly higher levels of melatonin than in the control group. The results showed that the mitochondria of porcine oocytes synthesized melatonin during oocyte maturation.

During maturation, the oocytes demand substantial amounts of ATP for their continuous transcriptional and translational activities. Mitochondria are the primary source of ATP production and sufficient numbers of functional mitochondria are critical for oocyte maturation. Therefore, the quality of oocytes is positively related to mitochondrial DNA copy number and ATP content. Under physiological conditions, the copy number of mitochondrial DNA, as well as mitochondrial distribution, significantly improved during oocyte maturation. However, in vitro maturation (IVM) may result in altered mitochondrial morphology and the expression of genes related to mitochondrial function [40]. In this study, we found that 5-HT supplementation significantly increased melatonin production; this elevated melatonin production increased the number of mitochondria, thereby promoting the uniform distribution of mitochondria, and thus increasing the ATP content in oocytes. These activities of melatonin were probably mediated by its receptor since the melatonin receptor inhibitor Luzindole blunted all these activities. To further identify the effects of melatonin on mitochondrial function and oocyte maturation, the *AANAT* was silenced by siAANAT, which can significantly reduce melatonin production. The siAAMAT caused abnormal mitochondrial distribution and mitochondrial dysfunction, while melatonin supplementation corrected these abnormalities. The results showed that melatonin is necessary for mitochondrial function, oocyte maturation, and embryonic development.

To further prove that endogenously generated melatonin is involved in oocyte quality and its maturation, 5-HT was used to increase endogenous melatonin production. The results showed that 5-HT supplementation had similar effects to melatonin supplementation. Melatonin is a mitochondrial-targeted antioxidant and it can upregulate the expression of *SIRT3* and *SOD1* [41]. Melatonin also protects against mitochondrial depletion and the energy deficiency caused by environmental toxin exposure by activating the *SIRT1/PGC-1α* pathway. These activities of melatonin promote mitochondrial biogenesis, suggesting that melatonin can be used in early embryonic development to counteract the state of mitochondrial deficiency [42]. In this study, we found that 5-HT significantly upregulated the expression level of SIRT1 after siAANAT, indicating that this effect may not involve melatonin, but instead, melatonin metabolites (see below). He et al. reported that melatonin can reduce the mitochondrial membrane potential, resulting in the quiescence of mitochondrial respiration and in maintaining a state of low metabolism [43]. However, our results found that melatonin increased the mitochondrial membrane potential, which may be due to a compensatory mechanism caused by the in vitro culture environment, in which there was an energy supply shortage.

**Figure 6:**
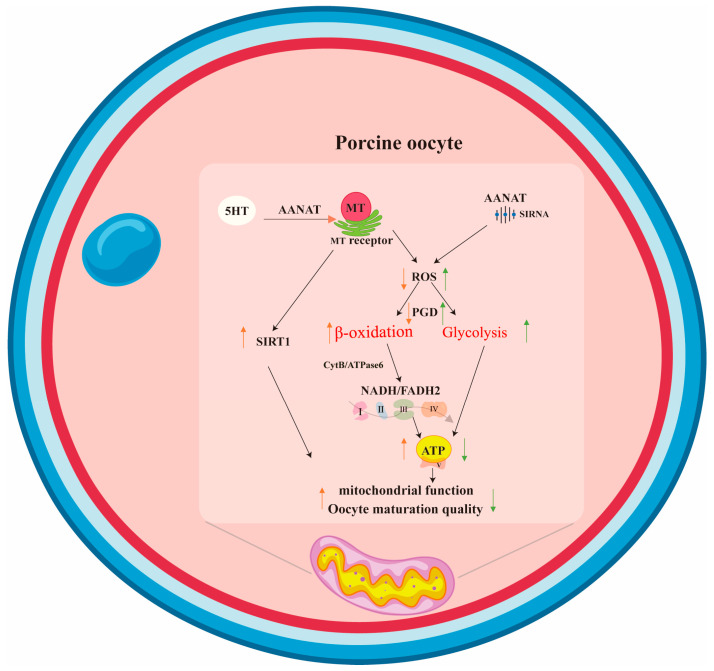
The potential pathways of porcine oocyte-synthesized melatonin and their effect on oocyte quality and maturation.

It has been reported that 5-HT and its receptors were present in mouse and human oocytes and cumulus cells [44,45,46], indicating their involvement in mammalian reproductive activity, including normal embryonic development. Other studies have reported that 5-HT administration caused blastocyst cell apoptosis and a decrease in blastocyst cell number and blastocyst rate [45,47]. The administration of 5-HT at a concentration of 10^−4^ M inhibited the maturation of porcine oocytes by inhibiting the synthesis of estradiol in granulosa cells, while its antagonists also inhibited mouse embryonic development and even caused embryonic development blockage at high concentrations under in vitro conditions [48]. In the current study, we speculated that some activities of 5-HT regarding oocytes and embryonic development were mediated by its metabolite, melatonin. The evidence obtained from this study strongly supported our speculation. Our results showed that 5-HT supplementation not only upregulated the expression of *AANAT* but also increased melatonin production in oocytes. The increased melatonin level was positively associated with oocyte quality and embryonic development (Figure 6).

Traditionally, it was believed that energy metabolism in the COCs was in collaboration among the cells. The cumulus cells are responsible for metabolizing glucose to form pyruvate and lactate, then both of them are transported to the oocyte through gap junctions, and finally enter the TCA cycle to produce ATP in the oocytes [49,50,51]. However, the porcine COCs may prefer to use fatty acids as the energy source [52]. Fatty acids undergo beta oxidation to produce ATP [53]. The reduced abundance of *CPT1* impairs the transportation of fatty acids into mitochondria, leading to reduced β-oxidation. This will be compensated for by the elevation of glucose metabolism in porcine embryos, suggesting that fatty acid oxidation is prevalent in the alternative energy pathway of glucose metabolism in this cell [54]. Further evidence showed that siAANAT significantly upregulated the expression of the key genes of *PGD* and *LDHA,* related to the Warburg effect, to increase glycolytic activity, but with decreased ATP production in the *siAANAT* oocytes compared to the controls. Melatonin supplementation reduced these upregulated genes. The results indicate that melatonin may induce porcine oocytes to preferentially use lipids for fatty acid β-oxidation to provide energy for porcine oocyte maturation.

## 5. Conclusions

In summary, in this study, for the first time in the literature, we have identified that the mitochondria of porcine oocytes are major sites for melatonin biosynthesis. This mitochondrially originated melatonin reduces ROS, upregulates the expression of *SIRT1*, and increases the number of mitochondria and their uniform distribution, as well as their oxidative phosphorylation, thereby improving the maturation efficiency of porcine oocytes. The reduced melatonin level by si*AANAT* upregulates the expression of *PGD* and *LDHA,* thereby switching the mitochondrial oxidative phosphorylation to glycolysis and reducing the maturation efficiency of porcine oocytes. These abnormalities are counteracted by melatonin supplementation. All these effects of melatonin are at least partially mediated by its receptors since the non-selective melatonin receptor blocker, Luzindole, blunts these activities. The research results provide an experimental basis for further revealing the metabolic mode of melatonin in porcine oocyte maturation. If this observation is confirmed by others or in different mammalian species, it will provide new insights to treat human infertility and support the conservation of germplasm resources in animal husbandry.

## Figures and Tables

**Table 1:** Primer sequence.

Genes	Accession Number	Sequence (5′-3′)
ATPase6	NP_008639.1	F ACTCATTCACACCCACCACACA
		R CCTGCTGTAATGTGGCTGTCA
HIF1A	NM_001123124	F ATTTCCATCTCCTCCCCACGTA
		R ACTCAAAGCGACAGATAACACA
GLUT1	XM_021096908.1	F CATCGTCGTCGGCATCCT
		R GGTTCTCCTCATTGCGGTTG
AKT1	NM_001159776	F GGCCCAACACCTTCATCATCCG
		R ATCTCTCCTCCTGCCGCTTG
PGD	XM_003127557	F AGAACCTGCTCCTGGATGACT
		R ATCTCGTGTCTGTACCCGTCGT
LDHA	XM_013994501.2	F GAAAGCCGTCTTAATTTGGTC
		R TTCCAAGCCACATAGGTCA
SOD1	NM_001190422.1	F AAGATTCTGTGATCGCCTCT
		R ACTTCCAGCATTTCCCGTCT
NRF2	XM_013984303.2	F AGTCCAGAAACCAAACCGACA
		R AATCTGTGTTICCTGTTGCGTA
SIRT3	NM_001110057.1	F TTTCGCCAAGGAGCTGTACCC
		R CTCTCTCAAGCCCGTCGATG
ND1	NP_008634	F TAATCACAACACAAGAGCACA
		R CGGCTGCATATTCTACGTT
COX3	NP_008640.1	F TCAGAATATTACGAAGCACCA
		R ATCCGATGATTACGTGCAA
CyTB	NP_008646.1	F CCACCCCATATTAAACCAG
		R TACTAGGGCCAACACTCCA

## Data Availability

Correspondence and requests for materials should be addressed to Guoshi Liu.

## Abbreviations

ROS: reactive oxygen species; ATP: adenosine triphosphate; AANAT: arylalkylamine-N-acetyltransferase; 5HT: 5-hydroxytryptamine; ASMT: hydroxyindole-O-methyltransferase; IVF: in vitro fertilization; COCS: cumulus–oocyte complexes; IVC: in vitro culture; LC-MS/MS: liquid chromatography mass spectrometry–mass spectrometry; MI: metaphase I; MII: metaphase II; GV: germinal vesicle; GVBD: germinal vesicle breakdown; Mito: mitochondria; Lu: Luzindole; MT: melatonin; IBMX: isobutyl methylxanthine; NC: negative control; IVM: in vitro maturation.
